# Acceptability of Novel Life Logging Technology to Determine Context of Sedentary Behavior in Older Adults

**DOI:** 10.3934/publichealth.2016.1.158

**Published:** 2016-03-24

**Authors:** Juliet A Harvey, Dawn A Skelton, Sebastien F M Chastin

**Affiliations:** Glasgow Caledonian University, School of Health and Life Sciences, Institute of Allied Health Research, Glasgow, UK

**Keywords:** Sedentary, Lifelogging, Objective Monitoring, Older Adults

## Abstract

**Objective:**

Lifelogging, using body worn sensors (activity monitors and time lapse photography) has the potential to shed light on the context of sedentary behaviour. The objectives of this study were to examine the acceptability, to older adults, of using lifelogging technology and indicate its usefulness for understanding behaviour.

**Method:**

6 older adults (4 males, mean age: 68yrs) wore the equipment (ActivPAL™ and Vicon Revue™/SenseCam™) for 7 consecutive days during free-living activity. The older adults' perception of the lifelogging technology was assessed through semi-structured interviews, including a brief questionnaire (Likert scale), and reference to the researcher's diary.

**Results:**

Older adults in this study found the equipment acceptable to wear and it did not interfere with privacy, safety or create reactivity, but they reported problems with the actual technical functioning of the camera.

**Conclusion:**

This combination of sensors has good potential to provide lifelogging information on the context of sedentary behaviour.

## 1. Introduction

Sedentary behaviour is defined by a sitting/lying posture and low energy expenditure (< 1.5 METS) during waking hours and includes activities, such as watching television, computer use and reading [1]. Sedentary behaviour is independently linked to morbidity and mortality and influences successful ageing [2],[3]. In most high, and some middle to low, income countries, promoting active ageing to decrease the burden of chronic disease and increase quality of life in later life, has become a major public health focus [4]. National and international guidelines recommend both increasing physical activity and reducing sedentary behaviour in all age groups [5],[6].

Older adults are the most sedentary and inactive sector of the population [7],[8]. Generally, this section of society is underserved in physical activity research [9]. The lack of initiatives to promote technology use to reduce sedentary behaviour in older adults stems from many factors including a lack of information about the context of behaviour in this population [10],[11] and a perception that technology to support intervention is often deemed unsuitable for older adults [12].

Knowledge exists on broad types of sedentary behaviour via self-report (e.g. via questionnaire or interview), but specific information on the true context of the behaviour is limited to activities, such as self-report of TV watching or screen time, but there is little information on other circumstances of sitting, which account for large portions of an individual's day. Some of these may include activities that are necessary and cannot, or should not, be altered, such as eating, resting, social or entertainment events. Self-report is open to misinterpretation of questions, social desirability bias or recall bias. Self-report rarely goes beyond certain activities and the environment of the activity therefore, the true context of sedentary behaviour needs to be explored.

When measured objectively, higher levels of sedentary behaviour are routinely recorded when compared to subjective reporting [13]. Although objective measurement provides unambiguous time monitoring of sitting or low energy expenditure, there is no context to the behaviour. Therefore, developing measurement techniques to lifelog or collect information about the context of sedentary behaviour in older adults is crucial to allow for the appropriate development of interventions. In addition, recent studies have shown that behavioural feedback, using modern sensors and information technology, is rated by older adults as a main contributor to successful behaviour change [14].

Advances in wearable sensor technology now enable continuous tracking of health behaviour and provide the opportunity to address knowledge gaps highlighted [15]. Wearable time-lapse lifelogging cameras are becoming more ubiquitous, cheaper and can record, passively, the context of behaviour. Combining wearable cameras with objective activity monitors and interview of individuals has the potential to produce information that is likely to shed light on the ecological determinants of sedentary behaviour and provide effective mechanisms for accurate information and timely feedback, necessary when examining behaviour [16]. In contrast to algorithm based assessment of behaviour, a wearable camera, in combination with an objective monitor, provide a memory prompt to older adults to make recall of daily activities easier.

The images produced by lifelogging provide information on: environment, social interaction, the activity itself and the interlinking of behaviour patterns, i.e. for each event there is a very detailed picture of what is happening, where is it happening, when is it happening, what is happening at the same time, and who is around. Using the system as a prompt to aid the participant's recall, within an interview, it is possible to tease out critical elements of behaviour choice and learn why these choices were made by the individual. Taking this a step further, the lifelogging concept can be used as a behaviour change intervention by allowing the participant to reflect on behaviour and self-evaluate/monitor choices made, allowing more effective problem identification and goal setting. The lack of knowledge on the context of behaviour is a major obstacle to progress in both assessment and interventions of lifestyle behaviour change.

SenseCam™, later to become Vicon Revue™ (Version:3MP™, Oxford, UK), was the first commercially available wearable camera and has been used in previous research, particularly in memory studies [17],[18]. A non-functioning mock-up of the camera itself has been shown to be generally acceptable to adult users [19]. The Vicon Revue™ measures 2.8 x2.6 x0.7 inches with a weight of 3.3oz. Vicon Revue™ has rarely been used in combination with other devices in the older adult population. Smeaton et al. [20] coupled SenseCam™ with a fall detection system and Wilson et al. [21] coupled it with an activity vest. One recent paper has coupled a camera with an accelerometer, but has not considered acceptability [22]. Work has been published showing feasibility of coupling these devices with younger populations [18],[23],[24].

There is a need for this technology to be used in much larger scale studies and more diverse populations. This cannot be achieved unless there is good compliance and ease of use of the technology in older adults. This study aimed to examine the acceptability to older adults of using lifelogging technology (consisting of a body worn camera alongside an objective physical activity monitor) to observe the free-living environment, which would allow observation of the context of their sedentary behaviour.

## 2. Materials and method

### 2.1. Participants

Ethical approval was gained from Glasgow Caledonian University (GCU) Ethics Committee and initial laboratory reliability tests were carried out using both pieces of equipment. Invitation letters were sent out to members of the GCU older adult database (consisting of approximately 200 older adults in the Glasgow area), inviting participation from those who had not used body worn camera or activity monitors previously. Six older participants were recruited and gave informed consent to take part in the study and for their camera images (anonymised) to be used for the study, in peer reviewed journals and presentations. The participants met the following inclusion criteria: 65+ years of age; community dwelling; able to ambulate independently; able to give informed consent; with no allergy to adhesive tape (necessary for securing ActivPAL™).

### 2.2. Equipment

The ActivPAL™ activity monitor is a thigh mounted objective monitor of free-living activity. The monitor has been shown to be accurate for measurement of static and dynamic activity, posture and is regarded as a gold standard for detecting periods of sedentary behaviour [25]–[27]. It defines sedentary behaviour as sitting/lying, but cannot distinguish between the two postures. It has been extensively used in older adult populations with good compliance and acceptability [28],[29].

**Figure 1:**
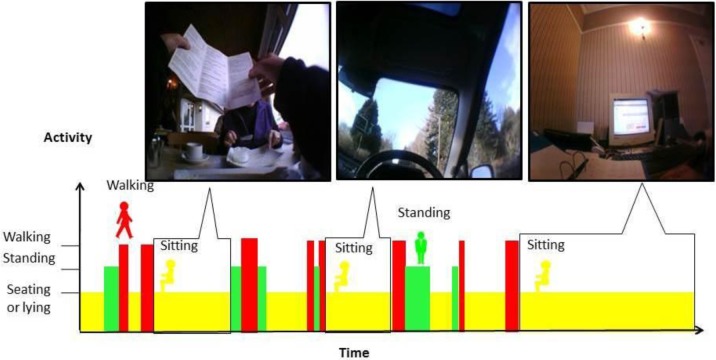
The concept of using activity monitoring and images in combination

Vicon Revue™ is a body worn time lapse camera, which passively records the context of the participant's free-living behaviour by collecting images when a change in environment is detected (e.g. movement, temperature, light). On average it collects 5 pictures per minute. A pictorial representation of how the device outputs could be coupled with ActivPAL™ data can be seen in Figure 1.

### 2.3. Protocol

The best research practice that has been put in place by previous authors was followed [17],[30],[31]. The participants were met at their preferred location (everyone chose their own home), where they received study instruction. They were requested to wear the equipment (ActivPAL™ and Vicon Revue™) for 7 consecutive days. Seven days of monitoring has been routinely recommended in activity monitoring studies to take account of weekend variation and has achieved 80% inter-class correlation [32]. The Vicon Revue™ was not to be exposed to water or worn at times deemed inappropriate and this was described in the information sheet (such as, public toilets, airports, banks, schools, changing areas, etc.). It was not to be worn whilst sleeping. When they entered a private dwelling they were instructed to ask permission of the occupants, this included their own home, where they sought permission of co-habitants. When the camera was not in use, a diary was kept by the participant to record the reason for turning the camera off, or not wearing the camera. The reasons for this were to evaluate if this may affect the overall evaluation of the data and to provide contextual information in the absence of the camera images. They also noted night-time sleeping.

The participants were given a short script explaining the use of the camera to show to others if they felt this was required. The 24hr telephone number of the principle researcher was also provided on the back of the camera in case of any issue with the study. Anyone could contact the researcher at any time and everyone was free to ask for any pictures to be deleted. Between visits, the camera unit was charged overnight by the participants as its battery capacity only allowed for one full day of recording.

The ActivPAL™ is protected by a waterproof pouch and attached to the leg using a hypoallergenic fixing tape. The ActivPAL™ was attached mid-thigh and was to be worn at all times for 7 days, including showering and bathing. The participant was instructed that if skin irritation occurred under the ActivPAL™, they were to remove it immediately, rinse the area with cold water and contact the researcher. The researcher kept a diary of issues raised over the study period, for example if a participant called with a query about turning on the camera.

On the second visit, the researcher retrieved the equipment (ActivPAL™ and Vicon Revue™) and the participants' were interviewed on the use of the equipment over the week. This interview was based on previous work by Wilson et al. [21]. The semi-structured face-to-face interview included completion of set of brief questions using a 5-point Likert scale and further comments (open-response) covering ease of use, privacy, reactivity, and safety, as shown in Table 1. As the study aimed to understand acceptability of the lifelogging equipment (both monitor and camera), the combined response the participants gave was recorded on a standard response sheet. The use of this style of questioning means the same information is collected from all respondents. The participants checked the response sheet to confirm that it was a true representation of their experience of the study. The final section of the interview asked the participants to make any further comment on the acceptability of using the camera. To reduce response bias the “reactivity” question was asked in reverse to the other questions. Following the interview, the equipment was then taken back to the university to be downloaded.

**Table 1:** Questions asked in the post study interview (Likert scale: 1-strongly agree to 5-strongly disagree, along with comments section).

Topic	Actual Question
Ease of Use	“The equipment was easy to use (camera & activity sensor)?”
Privacy	“The camera allowed sufficient privacy to you and those who you interact with?”
Reactivity	“Wearing the camera and sensor affected your normal day to day living?”
Safety	“Did you feel safe whilst wearing the camera and sensors?”

In the final session, within 2 weeks of the previous session, the participants had the opportunity to review their images, using Docherty Wearable Camera Browser [33], and delete any they selected. The ownership of the images remained with GCU and the participants were not permitted to print or have a copy of the images. This was to ensure that the images were not used for anything other than research purposes [32]. The aim of this paper is to explore the acceptability of lifelogging in older adults. *Supplementary material* is provided to demonstrate the application of its use in defining the context of prolonged sedentary behaviour (4 participants).

The same researcher collected the data and carried out the analysis. For the semi-structured interview data, the responses on the Likert scales were collated and presented graphically. The qualitative results of the structured interview were collated into themes, using thematic analysis. Finally, the researcher's diary was reviewed for the themes that emerged and a count was made of the number of reports under each theme and presented graphically.

## 3. Results

6 participants (4 male), mean age 68 (± 2.3) years, completed the study and the post study interview. They had an average body mass index of 25.6 (±5.2) kg/cm^2^, 5/6 were on 2 or more medications, 1/6 was a frequent faller, 2/6 has attended hospital in the last year, 2/6 were educated beyond high school level. Two participants did not review their images. The first, due to ill-health of a family member, declined to attend the final session. The second was unable to review due to technical difficulties with the equipment (ActivPAL™).

Four participants had full 7 day ActivPAL™ data sets, 1 participant had 6 (one non-wear day) and 1 had only 2 days valid wear (fault in unit), all participants wore the monitors for 7 days. The ActivPAL™ software allowed fast download, within minutes, and relatively easy analysis. It produces a data sheet for scientific analysis and a summary sheet that is user friendly, allowing for easy translation of the information to the lay person.

Vicon Revue™ images were collected from all participants' the mean wear time of the camera was 6.5/7 days. The images took an average of 2hrs 8mins (±1hr 32mins) to download onto a Laptop. The average number of images produced in the week was 21174.5 ± 2210.8 and the average file size was 5.6GB ± 0.7. The researcher reviewed the 127,000 images collected during the study, observing 0.7% were blank images (unintelligible) and 3.6% were frozen images (time and image number move on, but the image remains the same). An overview to check for image clarity takes about 60mins per participant, which would not be acceptable for most people. Automatic synchronisation of the images with the activity monitor output could not be achieved due to the loss of time on the camera which occurred every time it was reset.

**Figure 2:**
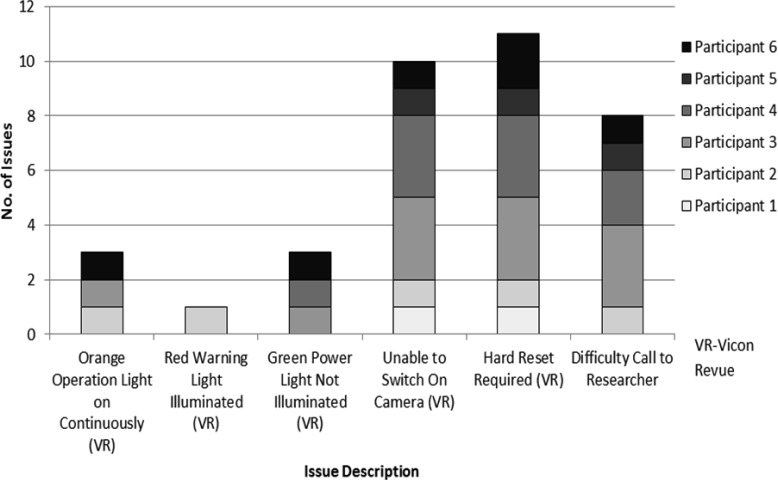
The participants' experience with the equipment reported to researcher (N = 6)

All 6 participants gave feedback on the study process, i.e., their experience with the equipment. When examining the researcher's diary, the participants reported varied problems with the Vicon Revue™ equipment and most participants made at least 1 call to the researcher during the study (mean calls: 1.3). There were no calls from anyone other than the participant. The most frequent problem was that they were unable to switch the camera on/power up after charging. This and other problems could only be resolved by carrying out a “hard reset” on the camera, this returns the camera to a default setting of a fixed time and date making it hard to time set the viewing of the images with the ActivPAL™ output. A summary of issues reported to the researcher can be found in Figure 2.

**Figure 3:**
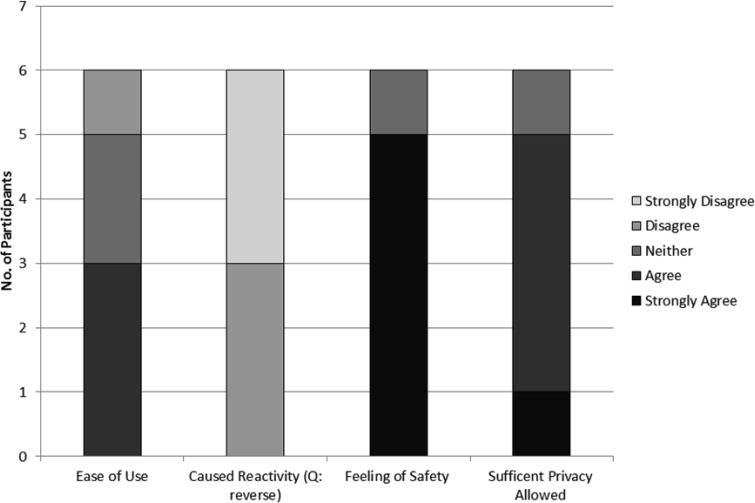
The participants' experience with the equipment, from interview (N = 6) (Likert scale: 1- strongly agree to 5-strongly disagree).

A summary of results of the interview are shown in Figure 3 and the themes of comments made by the participants are shown in Table 2. In general the actual wearing of the equipment was acceptable for the older adults participating in this study. There were no notable gender differences in acceptability in this small sample.

**Table 2:** Summary of comments from participants on participation in the study.

Theme	Participant Comment Summary
Use of Camera	The main problem was with the actual functioning of the camera – turning it on and off and power issues.Participant's generally found the equipment acceptable to wear.
Reactivity	The equipment has little effect on the participant's day to day life.They tended to forget they were wearing both pieces of equipment.
Safety	The participant's felt safe whilst using the equipment.
Privacy and Interaction with Others	The Vicon Revue allowed themselves and those whom they interacted with sufficient privacy (participant's perspective).Participant expected more reaction from others and those who they interacted with found the equipment to be acceptable.

### 3.1 Ease of Use

5 out of 6 participants agreed that the equipment was easy to use. Participants reported very few problems or concerns with the wearing of the Vicon Revue™ camera. The difficulty arose with the functioning of the camera. Five out of six participants reported some sort of functional difficulty (ensuring the camera was on, turning the camera on, loss of power, button getting stuck). One participant reported the camera getting in the way “…*when eating things such as a bowl of soup*” [P2] and with the ActivPAL™ “sometimes *activity monitor catches on underwear*” [P2]. Some participants [P1], [P3], [P6], found the camera to be unstable at times, such as when walking *“[the camera] swings and bangs on chest*” [P6], so it seems fixation of the camera caused some problems.

Reactivity:

When asked if wearing the sensors changed behaviour, all participants disagreed. This was confirmed by additional comments and 2 participants [P2] [P3] noted that they actually forgot they were wearing the devices “*My behaviour wasn't affected because I tended to forget the sensor was there*” [P3].

Safety:

All participants agreed that they felt safe when using the devices. One participant noted “*I didn't think about the safety point of view, I was never concerned that someone would want to steal it or it ensured protection as it was a camera.*” [P6]. Another noted an “*aware[ness] of the camera when wearing around children especially with the inquisitive nature of children*” [P4].

Privacy:

No issues reported on the comments section of the interview and the Likert scale responses were either neutral or agreeable that there were no user privacy issues. It would appear the participants' expectations of others reactions was greater than what was actually experienced - “*nobody objected to my wearing of the device* [camera]” [P5]. Only one participant [P1] noted “*one person was suspicious of the camera, but it was ok when I explained what it* [the camera] *was for*”. In fact, another participant [P4] noted a “*positive reaction from others*” with the camera and stated it “*provided a topic of conversation*”.

## 4. Discussion

It may be assumed that older adults would find lifelogging to be invasive and inappropriate for this age group, however, participants in this acceptability study generally found the camera and activity monitor to be acceptable to use. One major issue arose with the actual functioning of the camera, where the participant had to perform a hard reset to allow the camera to turn on during the study period. This was not only inconvenient for the participant, but affected the timestamp of the image. Some participants found that the camera was physically intrusive at certain times, i.e. getting in the way. The single lanyard strap does mean that the camera has opportunity to swing in a pendulum fashion. This is not only inconvenient for the participant, but affects the quality of the images.

These issues have been reported to the manufacturers and the Vicon Revue™ has undergone a major re-design into a simpler and more user friendly design, commercialising under the name of Autographer, which has a less cumbersome design and can be clipped to clothing as an alternative to use of a lanyard strap. They also recommend a limit of 12 hour of recording before download. This resolves the need for hard reset and will allow for faster processing of the data. Notably, new devices from other manufacturers, such as Memoto and Google Glass, have intrinsically older adult friendly designs.

Surprisingly, the older adults had very little reaction from others with regard to wearing the camera. The participants and the researcher had expected much more negative feedback. Perhaps with the technological advances over the past decade we have become accustomed to seeing people wearing/using all sorts of devices in their day to day life. It is conceivable that we have become accustomed to our lives being recorded via technology, such as CCTV. It was very positive that the participants did not feel that wearing of the camera affected their privacy or feelings of safety.

Kelly et al. [34], in a paper focusing on the ethical framework of the use of body worn cameras, have shown that as long as cameras are used appropriately, legal and ethical obligations can be upheld. In this study, no legal or ethical implications were found. This lifelogging system has the potential to be used in larger scale studies, as long as the recommendations around autonomy, voluntariness, beneficence and non-maleficence from Kelly et al. [34] are adhered to. With the explosion of social networking and use/misuse of images, it is conceivable that there may be a change in the law with regard to use of image capture systems, therefore protocols may have to be changed in this instance [34].

The images give context to the behaviour and allow researchers and participants to examine activities that the participant is engaging in. The images give information to the viewer as to their body position, thus helping to define whether a person is, for example, sitting or lying. Currently, ActivPAL™ is not able to do this independently. A major problem that arose in this study was that the two devices could not be automatically synchronised due to the loss of the time stamp on the Vicon Revue™, leading to considerable researcher time synchronising the data.

Gaining participant's perspectives in the description of the context of their behaviour is important when considering behaviour change, as this is the type of information required to help shape interventions in reducing sedentary behaviour. Information such as why they are engaging in a particular activity (for example, “*I am* watching *the TV here because I am resting after spending an hour swimming”*), will lead to appropriate and tailored interventions which do not attempt to intervene on valued sedentary time. The combination of sensors could also be used to help with behavioural change interventions by allowing the user to self-monitor and reflect on their behaviour in a very detailed way. The actual system could be set up in individual's homes to allow them to do this with minimal input from others. Although this study focuses on the acceptability of two lifelogging pieces of equipment (which have now been improved as a result of this study), in a small sample of older adults, it does suggest that older adults would be amenable to such technology in the context of sedentary behaviour or physical activity interventions. The ability to view the context of their behaviour allows an in depth understanding of context (supplementary data) which both researchers and older people find useful.

## 5. Conclusion

In conclusion, the combination of sensors for lifelogging will allow researchers to determine context of sedentary behaviour in a very detailed way. Older adults participating in this study generally found the equipment to be acceptable to wear, did not feel they interfered with privacy or safety, nor change their behaviour. However, the Vicon Revue™ had intermittent technical issues, in particular, its inability to keep time and date loss issues. For lifelogging the context of sedentary behaviour, there is great potential in the coupling of activity monitors and image capture systems for use in larger scale population studies.

## Figures and Tables

**Figure S1.**
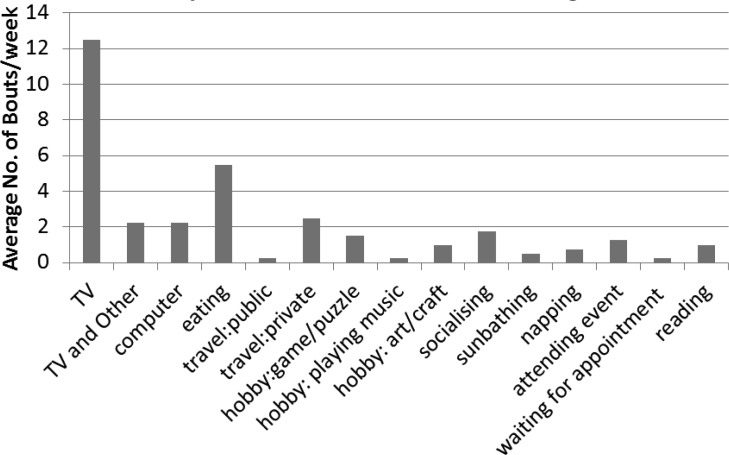
Average older adult's sedentary behaviour by activity over 30min provided as a per participant average over 7 days (N = 4).

**Figure S2.**
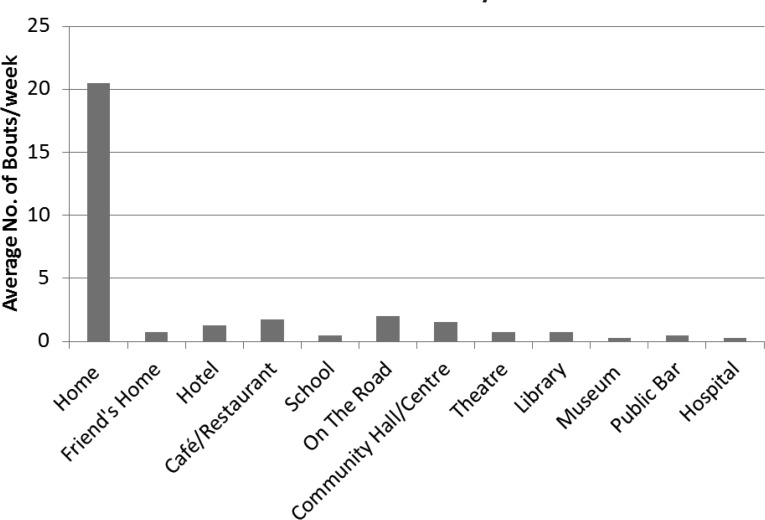
Location of older adults prolonged sedentary behaviour over 30min provided as a per participant avarage over 7 days (N = 4).

**Figure S3.**
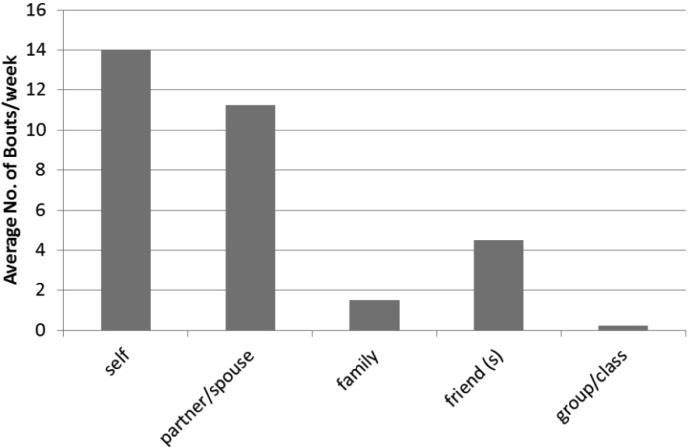
Social interaction when older adults engage in prolonged sedentary behaviour over 30mins provided as a participant average over 7 days (N = 4).

**Figure S4.**
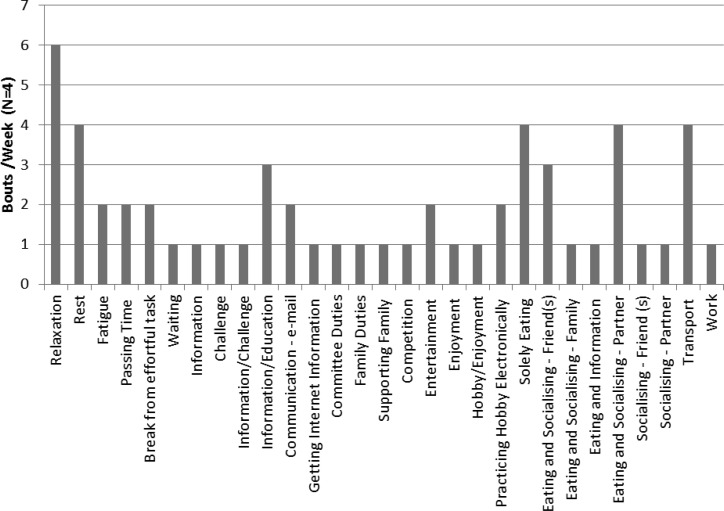
All 4 participants reasons for prolonged sedentary time (N = 4).

## Supplementary Material

### Illustration of Lifelogging Use: Context of Sedentary Behaviour

Analysis of the context of behaviour was conducted using the activity monitor outputs and day by day reflection of behaviour, where sedentary time was over 30mins. This time was selected as bouts of sedentary behaviour under 30min are probably not significant to health outcomes [1]. This was appropriate for a sub-group of 4 participants (Mean Age: 67.5 ± 1.3 years; 100% male).

In an average 24hrs period, this sample of older men were sitting or lying for 16.8 ± 2.3hrs, thus 70% of a 24hr day. When considering all sedentary time over 30min, excluding night-time sleeping, there were 32 bouts in a week of sedentary time, per person on average. The average bout length, once sitting over 30mins, was 51.1±6.55 mins. Considering the contextual information gained, TV viewing was the most common activity that engaged older adults in prolonged sitting, with an average of 15 bouts a week, but was not the only activity keeping older adults engaged in sitting (see Figure S1).

Prolonged periods of sedentary time tended to be in the evening, individuals tended to be at home and were most commonly on their own, as can be seem from Figure S2 and S3.

There was a diversity of reasons given by the participants for being sedentary for prolonged periods. The most frequently stated reasons for being sedentary were reasons relating to energy levels, such as rest, relaxation and fatigue. The second most common reason seems to be around socialising and spending time with friends and family. Interestingly, often co-existing with other reasons, eating is often a reason for prolonged sitting observed by participants. Details of the reasons can be seen in Figure S4.

Feedback on sedentary behaviour over 30mins was an example of how the data could be used. It was not the intention of this small scale acceptability study to be a representation of the behavioural habits of the general older population. Although not done in the present study, analysis of behaviour could also be derived from data produced by equipment in the absence of the actual participant using automated technology. This would be less of a burden for the researcher and participant and allow for larger numbers to be sampled, but some of the richness of the data would be lost. Automated techniques to recognise indicators of lifestyle behaviour, using the sensors within the camera unit, are being developed [2]. The actual coding of behaviour can be carried out manually by researchers, such as the protocol used by Kerr et al. [3]. The analysis could also be done automatically using image recognition software. However, where participants are not involved in the review of the images it is not possible to gain information about their thoughts or feeling around the event.

Clarification is still required on a taxonomy used when describing sedentary behaviour. Current work by Chastin et al. [4] shows promise for development of taxonomy to define the context of sedentary behaviour. This taxonomy would be very useful to allow the researcher to define behaviour derived from lifelogging data in a standardised way.

This small pilot study is limited by it sample size and gender distribution. However, findings are comparable to other published findings. Gardiner, et al. [5], also highlighted that older adults are most sedentary in the evening and defines TV viewing a major issue in sedentary behaviour in older adults. Leask et al. [6] indicated that screen time, in particular TV viewing, was responsible for periods of sitting. The older adults were usually on their own, at home when sitting bouts occurred.
